# Induced Pluripotent Stem Cells: Generation Strategy and Epigenetic Mystery behind Reprogramming

**DOI:** 10.1155/2016/8415010

**Published:** 2016-01-05

**Authors:** Pengfei Ji, Sasicha Manupipatpong, Nina Xie, Yujing Li

**Affiliations:** ^1^Institute of Genetics and Development Biology, Chinese Academy of Sciences, Beijing 100101, China; ^2^Department of Human Genetics, Emory University School of Medicine, 615 Michael Street, Atlanta, GA 30322, USA; ^3^Department of Neurology, Xiangya Hospital, Central South University, Changsha, Hunan 410008, China

## Abstract

Possessing the ability of self-renewal with immortalization and potential for differentiation into different cell types, stem cells, particularly embryonic stem cells (ESC), have attracted significant attention since their discovery. As ESC research has played an essential role in developing our understanding of the mechanisms underlying reproduction, development, and cell (de)differentiation, significant efforts have been made in the biomedical study of ESC in recent decades. However, such studies of ESC have been hampered by the ethical issues and technological challenges surrounding them, therefore dramatically inhibiting the potential applications of ESC in basic biomedical studies and clinical medicine. Induced pluripotent stem cells (iPSCs), generated from the reprogrammed somatic cells, share similar characteristics including but not limited to the morphology and growth of ESC, self-renewal, and potential differentiation into various cell types. The discovery of the iPSC, unhindered by the aforementioned limitations of ESC, introduces a viable alternative to ESC. More importantly, the applications of iPSC in the development of disease models such as neurodegenerative disorders greatly enhance our understanding of the pathogenesis of such diseases and also facilitate the development of clinical therapeutic strategies using iPSC generated from patient somatic cells to avoid an immune rejection. In this review, we highlight the advances in iPSCs generation methods as well as the mechanisms behind their reprogramming. We also discuss future perspectives for the development of iPSC generation methods with higher efficiency and safety.

## 1. Introduction

Due to their characteristic pluripotency, stem cells have the capacity to unveil the mystery behind reproduction, regeneration, and (de)differentiation, rendering stem cell—in particular, embryonic stem cell (ESC)—research essential for the development of a fundamental understanding of biomedical mechanisms and the discovery of clinical therapeutic strategies [1]. However, stem cell research has suffered setbacks due to ethical controversy, resource limitation, and technological barriers, hindering its biomedical research and clinical applications for regeneration medicine and therapy. To overcome these limitations, biologically similar alternatives that can bypass the ethical issues surrounding stem cells are essential. Significant efforts in this regard have led to the generation of induced pluripotent stem cells, an important advancement in biomedical research. Specifically, iPSC has been applied for development of disease models for neurodegenerative disorders amongst others, greatly enhancing our understanding of the pathogenesis of such diseases, as well as allowing for the development of clinical therapeutic strategies using iPSC from patient somatic cells. As such, the research advances in neurodegenerative disease models have been well reviewed [2–5].

iPSC was initially generated by reactivating nuclear reprogramming factors to reverse differentiated cells into a reprogramming state [6–8], maintaining the abilities of self-renewal and potential differentiation into various cell types. iPSC, like ESCs, can differentiate into nearly all the cell types in the organism from which they originated, shedding light on cell-based therapies and regenerative medicine to which patient-specific iPSC could be applied in order to regenerate tissues or organs destroyed by injury, degenerative diseases, aging, or cancer while avoiding rejection by the host's immune system. This method is undoubtedly a milestone for stem cell research, as iPSC has been and will continue to be the primary substitute for or perhaps even surpass ESCs in their ability to serve as a tool to uncover the mystery behind differentiation.

Although an increasing number of groups thereafter have made significant efforts in the generation of iPSC from a variety of somatic cell populations, available information about the genome-wide epigenetic alterations that somatic cells must undergo to become fully reprogrammed remains limited. In addition, some concerns about the current procedures, particularly the insufficient efficiency and specificity required for clinical application, remain. Thus, a better understanding of the downstream events following the activation of silenced master reprogramming factors could provide essential information to aid in the development of patient-specific iPSC lines in a faster and safer way. In this review, recent advances in iPSC generation strategies and the detailed mechanisms that underlie reprogramming are highlighted, and future perspectives are discussed.

## 2. Technological Advances in iPSC Generation

In addition to efficiency and specificity concerns with regard to iPSC generation methods, there has been a concern over the virus based reprogramming as it may integrate unwanted vector fragments into iPSC genome, given that the Yamanaka factors such as Oct4, Sox2, Myc, and Klf4 (OSMK) are introduced into the fibroblast cells with the help of a virus. This would affect the clinical application of derived iPSC as it introduces the possibility of negative effects on the biological properties of iPSC and increases the likelihood of malignant transformation. Indeed, recent study showed that reactivation of viral genes integrated in host genome during differentiation of the reprogrammed iPSC leads to tumorigenesis [9]. To overcome the shortcomings conferred by the traditional methods, efforts have been made to address the efficiency and safety issues as described below.

### 2.1. Epigenetic Operation

To tackle the problem of low efficiency, chemical as well as epigenetic approaches have been adopted with the aim of enhancing iPSC generation efficiency [10–12]. Epigenetic regulations drive the reprogramming of histone methylation and acetylation levels. As some histone methyltransferases have been acknowledged to play significant roles in the inhibition of reprogramming efficiency via methylation, it is logical to speculate that repression of histone methyltransferase expression or inhibition of its activities would enhance reprogramming efficiency. Indeed, shRNA-based knockdown of H3K9 histone methyltransferase DOT1L, leading to significant elevation of NANOG and LIN28 levels at an early stage of reprogramming, dramatically promotes the generation of iPSC colonies even without the overexpression of KLF4 and c-Myc [13]. A related mechanism is DOT1L repression-mediated loss of H3K9me2 in genes involved in mesenchymal to epithelial transition (MET). Accordingly, independent studies show that the overexpression of demethylase Kdm4b, along with a deficiency in H3K9 methyltransferases Ehmt1, Ehmt2, and Setdb1, or heterochromatin protein-1*γ* (Cbx3), a protein known to recognize H3K9 methylation, could significantly promote reprogramming [14, 15].

Paradoxically, JMJD3, a histone H3K27 demethylase [16] expected to enhance reprogramming efficiency, instead represses reprogramming [17] through two potential pathways. The first is demethylase dependent; by increasing the demethylation of H3K27me3 at Ink4a/Arf loci, JMJD3 elevates expression levels [18]. The importance of this is well evidenced by the fact that knockdown or deletion of Ink4a/Arf drastically increases reprogramming efficiency. The second potential pathway is demethylase-independent degradation and ubiquitination of PHF20, which is required for reprogramming [17, 19].

Changes affecting the dynamic balance between acetylation and deacetylation may also affect reprogramming, as is evidenced by the effects of several core members of nucleosome remodeling and deacetylation (NuRD) repressor complexes on reprogramming efficiency. Serving as a core component in Methyl-CpG Binding Domain Protein 3 (Mbd3) NuRD repressor complexes, Mbd3 can interact with core reprogramming factors (OSKM) and assemble directly with Mbd3/NuRD to recruit the repressor complex to downstream OSKM target genes. As would be expected, Mbd3 depletion was capable of significantly enhancing the reprogramming efficiency of human and mouse fibroblast cells to near 100% within seven days [20], with the only concern being the quality of the induced iPSCs in the absence of Mbd3. It has been acknowledged that protein kinases make significant contributions to signal transduction in eukaryotic cells [21], suggesting their potential role in regulating somatic reprogramming. To this end, kinome-wide RNAi-based screening has been performed to identify the specific protein kinases that regulate reprogramming efficiency [22]. Among the 59 of kinases serving as potential barriers to reprogramming, serine/threonine kinases TESK1 and LIMK2 have been further tested to confirm their roles in MET during mouse embryonic fibroblast (MEF) reprogramming. Furthermore, TESK1 deficiency in human fibroblasts could significantly enhance reprogramming efficiency [22].

### 2.2. MicroRNA Manipulation

MicroRNA has been acknowledged to function as essential regulators for gene expression in almost all metabolic pathways, suggesting their potential involvement in the regulation of the nuclear reprogramming (Figure 3), and providing insight into means of enhancing reprogramming efficiency by alteration of miRNA expression levels. Certain microRNA, such as miR29b, directly target mRNA coding for several enzymes responsible for the methylation of cytosine (C) and demethylation of 5-methylcytosine (5-mC), mediated by 5-hydroxymethylcytosine (5-hmC) [23]. As a balance of 5-mC and 5-hmC has been essentially linked to somatic reprogramming [24], this suggests the regulatory functions of miR-29b in this regard. The miR-290–295 clusters, 2.2-kb region on chromosome 7 [25], constituting over 70% of the entire miRNA population in mouse ESCs and the most abundant miRNA family in ESCs, have been believed to be important regulators for the ESC-specific cell cycle. The miRNA members in the cluster such as miR-291-3p, miR-294, and miR-295 had the capacity to enhance Klf4-, Oct4-, and Sox2-mediated reprogramming efficiency, although they were unable to further promote pluripotency efficiency in the presence of cMyc. Further study shows that the miRNA is downstream effector of cMyc [26]. More excitingly, overexpression of the miR302/367 cluster alone could enhance the reprogramming of mouse and human somatic cells to an iPSC state much more rapidly and efficiently than endogenous overexpression of the master transcription factors Oct4/Sox2/Klf4/Myc. iPSCs generated from mouse and human somatic cells via the overexpression of miR302/367 display similar characteristics to the ones from the conventional reprogramming factors, from pluripotency marking to teratoma formation [27]. More and more miRNA which regulated reprogramming had been identified, for example, three miRNA clusters, miR-17~92, miR-106b~25, and miR-106a~363, which have the ability to significantly enhance the induction efficiency at early reprogramming stages. Furthermore, miR-93 and miR-106b share very similar seed regions and dramatically promote iPSC induction, resulting in mesenchymal to epithelial transition (MET) at the initiation stage of reprogramming. More interesting is the capability of these miRNA-mediated iPSC clones to reach a fully reprogrammed state. Further study shows that the miRNA functions as reprogramming enhancer by targeting p21 and TGF-*β* receptor II, as is evidenced by the fact that siRNA based knockdown of both targets significantly increases iPSC induction efficiency. Another mechanism for the enhancement of miRNA based reprogramming efficiency is the regulation of cell cycle-related genes [28, 29].

### 2.3. Activation of Core Factors for Reprogramming

Although the native forms of core factors have been widely employed in iPSC generation, their relatively low transactivation activity remains a barrier for somatic cell reprogramming [30]. Recent studies have shown that the modification of OCT4, SOX2, and NANOG provides a new approach to overcoming these barriers [30, 31]. The yes-associated protein (YAP) has been demonstrated to be a transcriptional coactivator with a potent transactivation domain (TAD) in the C-terminal region; ectopic expression of YAP promotes cell growth and induces tumor formation [19, 32]. In addition, YAP also plays a critical role in the maintenance of stem cell pluripotency [33].

To enhance iPSC generation efficiency, the Oct4, Sox2, Nanog, and Klf4 (OSNK) reprogramming factors were engineered such that the transactivation domain of YAP is fused to defined factors labeled as OySyNyK. The efficiency of OySyNyK-induced iPSC generation is dramatically enhanced due to these modifications (about 100-fold greater efficiency relative to that of the wild-type OSNK-induced iPSCs). Furthermore, the initiation of reprogramming by OySyNyK is much faster, usually occurring within 24 hours. To understand the mechanism underlying this enhanced reprogramming, an epigenetic study was performed, the results of which indicated that the engineered reprogramming factors significantly increase the expression level of one member, namely, Tet1, of the ten-eleven translocation proteins (TETs, Tet1, Tet2, and Tet3 in the genome of mammalian cells) at the early reprogramming stage and also produce a marked increase in 5-hydroxymethylcytosine (5-hmC) levels, collectively suggesting that the engineered reprogramming factors collaborate with TETs to regulate 5-hmC mediated epigenetic control of somatic reprogramming [12].

### 2.4. Elimination of the Unwanted Virus Vector Parts in iPSCs

The integration of unwanted vector fragments into the iPSC genome can adversely affect the clinical applications of iPSCs in therapy. Thus, the improvement of nonviral and integration-free alternative methods to eradicate the safety issues currently associated with iPSCs has been a goal since the early stages of iPSC development.

#### 2.4.1. Complete Removal of the Viral Vector in Cell Reprogramming

The initial approach for the removal of the unwanted viral vector was to combine a lentiviral vector with Cre to excise the reprogramming vectors flanked by loxP sites using transiently expressed Cre-recombinase. Although a large part of the lentiviral vector flanked by loxP sites can be removed, a small part of the vector DNA external to the loxP sites most probably still remains integrated. In addition, this strategy is difficult to operate and time consuming.

In another attempt to rectify this issue of viral vector integration, the PiggyBac (PB) transposon system has been used to efficiently integrate the construct harboring core factor genes into TTAA sites in the target genome. The inserted PiggyBac vector can be excised by PB transposase in a footprint-free removal [34–36].

#### 2.4.2. Nonviral Methods

Nonviral methods have also been developed, such as the utilization of episomal and minicircle vectors [37, 38], adenoviral vectors [39], and Sendai vectors [40–42], and these special nonviral vectors have been demonstrated to enhance the reprogramming efficiency without introduction of viral components into the iPSC genome. As episomal DNA vectors with smaller molecular size free of bacterial plasmid DNA backbone, minicircles are designed for circular expression cassettes by significantly enhancing the transfection efficiency and offering over a period of weeks expression instead of only for several days conferred by standard plasmid vectors. Since the sequences within the bacterial plasmid backbone harbor the signals for methylation and transgene silencing, the minicircles based transfections can overcome the short period expression conferred by traditional transient transfections of plasmids.

Compared to other virus vectors such as lentivirus vectors, adenovirus vectors possess many advantages such as conferring the very efficient nuclear entry and low pathogenicity for humans, transducing large genes of more than 30 kb, avoiding integration into the host cell genome, targeting cell specificity, and maintaining long-term expression of transgenes. As such, the adenovirus vectors have been developed as popular gene delivery vehicles in a wide range of transduction for different cell types, particularly for quiescent and differentiated cells in basic biomedical research, clinical applications such as gene therapy, and industrial applications such as vaccine development.

Different from all the conventional DNA vectors that so far have been extensively applied, Sendai virus (SeV) vector is a cytoplasmic RNA vector with RNA genome, a material chemically different from the patient's genome DNA. Thus, since the SeV vector replicates its genome exclusively in the cytoplasm instead of entering cell nucleus, it overcomes the fundamental risks in host cell chromosomal alteration caused by integration of DNA vectors into chromosomes or genetic recombination. Additionally, the SeV vector could produce protein in large quantity in the host cells. Given so many advantages over the conventional DNA vectors, SeV has been successfully applied in clinical therapy as well as basic biological research including iPSC generation.

To absolutely exclude virus vectors, electroporation of the constructs with nucleofection into somatic cells has become an alternative also [43].

#### 2.4.3. Integration-Free Method: Modified mRNA Strategy

This strategy is based on the administration of engineered mRNA coding for the core reprogramming factors (ONSMK) to avoid innate antiviral responses and has been proven to be significantly more efficient compared to the previously established protocols in reprogramming human fibroblast cells to generate RNA-induced pluripotent stem cells (RiPSCs) [44]. In addition to fibroblast cells, this technique has also been applied to iPSC generation from bone marrow-derived mesenchymal stromal cells (BM-MSCs). However, although this strategy bears an advantage in that it avoids involving transgenes, its low efficiency remains a big concern.

#### 2.4.4. DNA/RNA-Free Strategy

Besides the methods utilizing engineered mRNA (Section 2.4.3) and small molecules as described in Section 2.4, integration-free strategies, such as the treatment of somatic cells with purified core reprogramming factor proteins, significantly enhance reprogramming efficiency [45, 46].

### 2.5. Chemical Approach to Improving Reprogramming Efficiency

Theoretically, any molecules that target core epigenetic enzymes to alter the dynamic balance of methylation/demethylation could be potential candidates for enhancing or inhibiting somatic reprogramming efficiency (Figure 3). As expected, several small molecules have been identified to function as inhibitors of histone demethylases, such as BIX-01294, RG108, parnate, 5-azacytidine, or 3 histone deacetylase inhibitors (suberoylanilide hydroxamic acid, trichostatin A, and valproic acid). These compounds enhance reprogramming efficiency either individually or in collaboration with the transduction of certain core reprogramming factors by reducing the methylation level of H3K9mono-Me, H3K9di-Me, or the L-calcium channel agonist Bayk8644 [47–54]. In particular, the incomplete epigenetic reprogramming attributed to the epigenetic memory of original cell-type-specific genes may contribute to the subtle difference between iPSCs and ESCs and even among iPSC clones [55–61], leading to low quality iPSCs with limited clinical applications. In order to elevate iPSC quality, epigenetic memory must be largely erased, where 5-azacytidine and trichostatin A could efficiently function as erasers [58]. However, possible off-target effects may lower the viability of iPSC treatment with epigenetic memory erasers.

Altogether, recent studies suggest that small molecules which function to alter the dynamic balance between methylation and demethylation—either individually or collaboratively—could significantly enhance reprogramming and largely erase epigenetic memory in cell-specific genes retained in iPSCs, leading to a substantial improvement in the quality of reprogrammed iPSCs [58, 60, 61]. The advantage of the small molecule-based reprogramming is that no genetic engineering is necessary, avoiding the integration of the unwanted virus vector sequences as well as the side effects caused by transgenes.

However, its shortcomings cannot be ignored, one of which is that the off-target effects may adversely affect quality of the iPSCs generated.

In summary, methods have been developed to remove the unwanted virus vector sequences or exclude use of viruses altogether in virus-free and integration- or transgene-free methods. However, although integration-free methods are within the realm of possibility, these strategies still share the same shortcomings, one of which is extremely low reprogramming efficiency relative to lentiviral vector-mediated strategies. One more issue needs attention for comparison of the reprogramming efficiency mediated by modified mRNA and viral vector strategies such as lentiviral vector. Although the modified mRNA confers the lower induction efficiency relative to the viral vector, the percentage of the normal iPSC generated by this strategy is higher than that by the lentiviral vector due to immune response as well as other adverse factors such as genome instability and chromosomal variation caused by viral vector integration into the chromosome of the iPSC.

### 2.6. Automated, High-Throughput Derivation, Characterization, and Differentiation of iPSCs

For large scale generation and further differentiation of iPSCs into special tissues to be used in regeneration medicine for therapeutic purposes, an automatic platform was established starting from fibroblast cell preparation [62]. Since this strategy is a robot-based platform combining many related protocols for cell isolation, culture, distribution, induction of reprogramming with modified mRNA delivery, and differentiation of iPSCs, very limited manual intervention was employed. It was proven that using this platform high-quality and stable iPSCs could be induced with less line-to-line variation than is found in those generated via conventional strategies. Although this combined automatic platform would significantly contribute to iPSC-based regeneration medicine in the long run, high demands with regard to the equipment necessary hamper its application at present.

## 3. Mechanisms behind Somatic Reprogramming

### 3.1. Epigenetic Regulation at the Chromatin Level

Not all cells that gain expression of the core reprogramming factors are pluripotent, though they may be self-renewing, because some of them are trapped in a state of partial reprogramming [47, 48, 63]. To understand the barriers to reprogramming, epigenetic identification was carried out to determine levels of histone and DNA modification in partially reprogrammed cells, fully reprogrammed cells, ESCs, and starting somatic cells. Epigenetic marks were found to be altered genome-wide, leading to reactivation of the core pluripotency genes and large scale 5-mC demethylation [49]. When the somatic cells are treated with inhibitors of HDACs, DNMTs, and the G9a methyltransferase, reprogramming efficiency is significantly enhanced [47, 64–66], suggesting the regulatory role of epigenetic modifiers in reprogramming. Indeed, many of the epigenetic regulators are directly recruited by the reprogramming factors to stimulate the expression of downstream pluripotency genes during iPSC generation, and chromatin remodelers serve as the key components of the interactome between the epigenetic regulators, core factors, and pluripotency genes. Some modulators, such as Smarca4/Brg1 and Smarcc1/BAF155 AS, the ESC-specific BAF (esBAF) components, INO80, Wdr5, and Mbd3, can physically interact with or be recruited by some or all of the OSKM master factors, enhancing the binding of these master reprogramming factors to promoters, leading to either an increase of activation-associated markers H3K4me3 and H3K9 acetylation (H3K9ac) at target genes, or the recruitment of RNA polymerase II to promote the expression of their target genes [67–75].

Modifications of both histones and genomic DNA have been essentially linked to the regulation of master transcription factor-mediated reprogramming. At the histone level, a balance between the enzymes responsible for methylation and those responsible for demethylation, such as methyltransferases and demethylases, determines the global H3K9me3 levels during reprogramming. Genome regions occupied by H3K9me3, a heterochromatic histone mark, have been identified to efficiently prevent the binding of master transcription factors in both human and mouse fibroblasts, serving as major roadblocks during reprogramming [76–78].

Accordingly, Cbx3, a reader of H3K9me3, and H3K9 methyltransferases (Ehmt1 and Ehmt2) have been detected in the Nanog protein complexes in mouse ESCs, suggesting that the Nanog autorepression mechanism is mediated by the recruitment of these readers and methyltransferases by Nanog in the cells remaining in a nonreprogramming state [78–80].

### 3.2. Epigenetic Regulation at the Genomic DNA Level

At the genomic DNA level is a situation similar to that at the chromatin level—a balance between DNA methylation and demethylation determines the dynamic status of the somatic cell, leading it towards either reprogramming or remaining differentiated. Again, this balance is also regulated by DNA methyltransferases and demethylases. The demethylation of the promoter regions of the genes conferring pluripotency has become a prerequisite for epigenetic somatic reprogramming [81] and most probably occurs during posthistone modification [82].

More and more evidence is available to contribute to an understanding of the mechanisms of passive and active DNA demethylation. However, these two mechanisms do not contribute equally to the dynamic regulation of demethylation. The passive mechanism renders the automatic and gradual loss of methylation during cell cycles possible. In contrast, several enzymes are believed to be involved in the active mechanisms for demethylation, including activation-induced cytidine deaminase (AID) [83], TET [84], and thymine DNA glycosylase (TDG) [12, 24, 85–87]. AID is responsible for the deamination of 5-mC to thymine, leading to cytosine exchange and demethylation and thus subjecting it to DNA repair pathways. However, the contribution of AID-mediated active demethylation to nuclear reprogramming remains controversial due to inconsistent experimental results [83, 88, 89].

**Figure 1:**
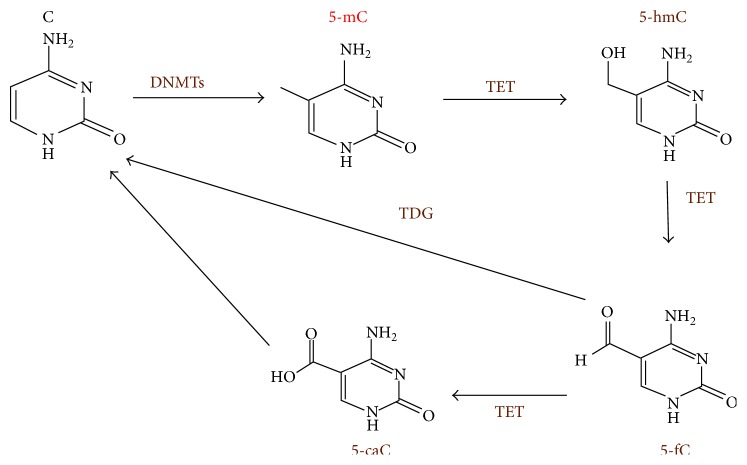
Base methylation at DNA levels. Cytosine (C) is methylated to methylcytosine (5-mC) by methyltransferases (DNMTs), and the 5-mC is oxidized or hydroxylated by ten-eleven translocation proteins (TETs) to 5-hydroxymethylcytosine (5-hmC). And the 5-hmC is believed to be mediator for demethylation 5-mC to C through serial steps: 5-mC is further oxidized by TETs to generate 5-formal C (5-fC) and 5-carboxy-C (5-caC), and finally 5-fC and 5-caC are converted to regular C catalyzed by thymidine DNA glycosylase (TDG). Methylation of adenosine at 6 position (6mA) has been also detected in the genomes of mammalian,* Chlamydomonas*,* Drosophila*, and* C. elegance* cells. TETs or TET analogues are responsible for generation of the 6mA.

Probably the most important mechanism for active DNA demethylation during induced pluripotency is the conversion of methylcytosine (5-mC) to 5-hydroxymethylcytosine (5-hmC) catalyzed by TET family (TET1, TET2, and TET3 in mammals). Once generated, the 5-hmC has to face several fates, either being directly converted into cytosine (C) through mechanisms involving the base excision repair pathway or sequentially becoming 5-fC and 5-caC to be finally converted into regular cytosine [90] (Figure 1).

Tet1 and Tet2 have been proven to facilitate cell reprogramming [84, 91]. To understand the roles that TET1 and TET2 play during somatic reprogramming, coimmunoprecipitation (Co-IP) and chromatin immunoprecipitation (ChIP) using antibodies raised against the TETs and master reprogramming factors were conducted. The results showed that TET1 and TET2 physically interact with NANOG, SOX2, and OCT4 at the protein level and that the master reprogramming factors such as NANOG, SOX2, and OCT4 recruit TET1 and TET2 to the key target genes to oxidize 5-mC to 5-hmC and then to cytosine either directly or indirectly [12, 84, 85].

Although knockdown of all three Tet genes in ESCs seems to confer ESCs normal self-renewal and pluripotency, deletion of Tet1 slightly enhances the reprogramming efficiency [92] and Tet3 deficiency had little effect, in contrast to the fact that inactivation of Tet2 reduced the reprogramming by 70%. While the double knockout of both Tet1 and Tet2 or Tet1 and Tet3 still does not significantly affect the reprogramming efficiency marked by the amount of the colonies with positive AP and SSEA1, deficiency of all three Tet genes completely abolished the reprogramming potential of MEFs. Further studies show that deletion of Tet2 from the Tet1 and Tet3 double knockout (DKO) or deficiency of Tet3 from Tet1 and Tet2 double KO MEFs completely inhibits the reprogramming, suggesting that the reprogramming deficiency of TKO MEFs could not be ascribed to inherent genomic or epigenomic alterations potentially arisen from the constitutive Tet deletion. Altogether this indicated that the Tet enzymes play essential roles in the key factors-driven reprogramming of somatic cells.

The complete abolition of the reprogramming of the Tet TKO MEFs has been linked to failing to undergo mesenchymal to epithelial Transition during MET [92]. Since the multistep process of the factor-driven reprogramming is initiated by MET [93, 94], any event affecting the MET process will be vital to the reprogramming. Indeed, Tet TKO MEFs showed no sign of epithelium-like morphological shift, in contrast to wild-type, individual, or double knockout of Tet. MEFs displayed a substantial MET, and the TKO MEFs significantly exhibited MET defect. The MET process in TKO MEFs could be rescued by ectopic expression of the active catalytic domains of the TETs, but not the inactive form of the full TETs, suggesting the TET might epigenetically function as 5-hmC mediated demethylation in MET process.

Applying the Tet-assisted bisulfite sequencing strategy by which the 5-mC and 5-hmC could be distinguished from each other [95], it was found that at early stage of the reprogramming the 5-hmC levels in the 5′ region of the promoters of miRNA such as miR-200s reached high percentage in WT MEFs, and strikingly the 5-hmC levels dramatically decreased in the Tet KO particularly in Tet2 KO and even completely abolished in the TKO MEFs [89]. Taken together, these indicated that the impaired oxidative demethylation of miRNA genes in Tet-deficient MEFs leads to inactivation of the miRNA such as miR-200s, miR-200a, miR-200b, miR-200c, miR-141, and miR-429 critically involved in both cancer metastasis and experimental cell reprogramming. And further evidences showed the inverse correlation between 5-hmC and 5-mC among promoters of the genes associated with cell adhesion, suggesting that the genes involved in MET serve as targets for Tet-catalyzed hydroxylation during the early phase of reprogramming [92].

**Figure 2:**
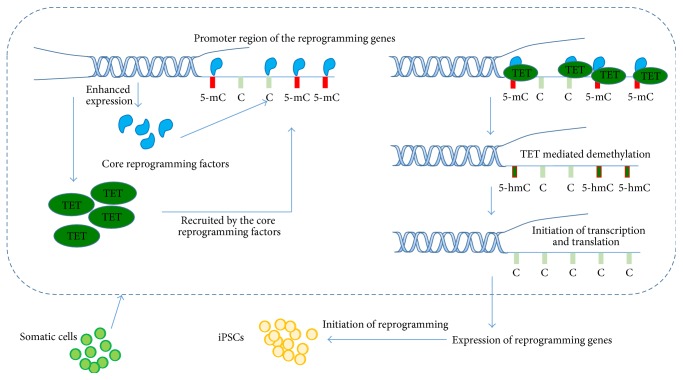
Enhanced expression of the core reprogramming factors and TETs switches the cell fate towards nucleic reprogramming to generate iPSCs. The TET enzymes are recruited by core transcription factors and localized to the methylated promoter regions of the downstream genes involved in the reprogramming. Then the TET oxidizes the 5-mC into 5-hydroxymethylcytosine (5-hmC) that serves as mediator of demethylation of the 5-mC, leading to demethylation of the promoter regions and expression of these genes and thereby initiation of the reprogramming.

**Figure 3:**
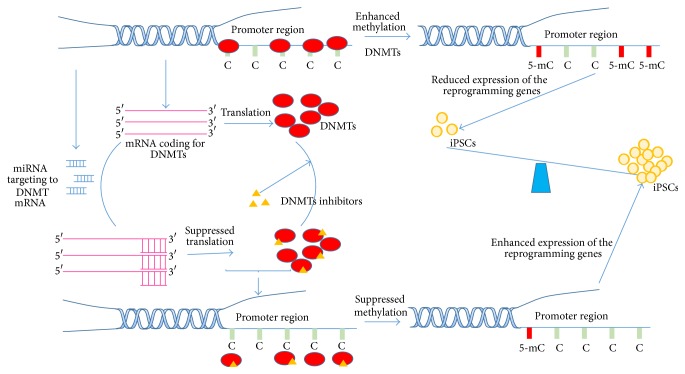
Regulation of the dynamic balance between methylation and demethylation by miRNA and small molecules functions as DNMTs inhibitors. Some miRNA targeted the mRNA coding for DNMTs and some small molecules function as inhibitors of DNMTs. Due to overexpression of some miRNA specifically targeting to DNMTs mRNA as well as treatment with DNMTs inhibitors, methylation is repressed, leading to dynamic alteration of methylation and demethylation balance and enhancing the reprogramming efficiency.

More interestingly, while both TET1 and TET2 are responsible for the conversion of 5-mC into 5-hmC, their roles in reprogramming are very different, especially in the presence of vitamin C [86]. While TET2 constitutively enhances the reprogramming regardless of vitamin C level, TET1 serves as a barrier to reprogramming by interfering with MET in the presence of vitamin C. In addition, TET2 but not TET1 can work together with Parp1 to balance the levels of 5-mC and 5-hmC. Besides Parp1, the DNA repair proteins that have been identified and linked to reprogramming include members in the XPC nucleotide excision repair complex. Unlike Parp1, the XPC family members are recruited by Oct4 and Sox2 to the Nanog and Oct4 promoters, working with TET2 to regulate reprogramming [96]. These findings suggest that various modulators required for reprogramming, such as TET1, TET2, and TET3, are recruited to specific DNA targets by core transcriptional factors to regulate the reprogramming state (Figure 2).

Besides the methylation of cytosine to form 5-mC, deoxyadenosine methylation has also been detected in the genomes of some eukaryotes, such as* Chlamydomonas, Drosophila,* and* C. elegans*, generating N6-methyldeoxyadenosine (6mA or m6A) [97–99]. Although the importance of the dynamic balance between the methylation and demethylation of adenosine in eukaryotes remains elusive, mammalian TET analogues in* Drosophila* (DMAD) and* C. elegans* (NMAD-1) function as erasers of 6mA methylation and play essential roles in reproduction and neuronal activities, further suggesting a TET- or TET analogue-mediated epigenetic regulation spectrum in eukaryotes.

### 3.3. Epigenetic Regulation at the Histone MacroH2A Level

Unlike TETs, which are guided by the core reprogramming factors and specifically localize to regions of the target genes to enhance active demethylation during reprogramming, some epigenetic regulators, such as macroH2A and peptidylarginine deiminase Padi4, globally rather than specifically affect the pluripotent state either negatively or positively [100–102]. MacroH2A, a histone variant, has a global adverse effect on reprogramming, evidenced by the fact that MacroH2A is depleted in pluripotent cells and enriched in differentiated cells, possibly to repress pluripotency factors, as well as that the removal of MacroH2A enhances the efficiency of reprogramming [98, 99]. In contrast, Padi4 plays positive roles in reprogramming by interfering with the binding of the histone H1 to nucleosomal DNA [99].

### 3.4. Nonepigenetic Regulation of Reprogramming Efficiency

In addition to the genome-wide epigenetic regulation of reprogramming, alterations of key components in some metabolic pathways such as p21 and p53, as well as some general transcriptional and translational apparatuses, also affect reprogramming efficiency. It has been shown that p21 can negatively affect reprogramming efficiency; consequently any alterations which enhance p21 transcription and translation would repress reprogramming [101]. Thus, it is logical to speculate that p53, which stimulates the transcription of p21, and eIF4E binding protein (4E-BPs), which enhances the translation of p21, could significantly inhibit reprogramming efficiency. This speculation has been confirmed by the fact that depletion of 4E-BPs in p53 deletion fibroblasts results in increased reprogramming efficiency compared to that in wild-type fibroblasts due to the reduced transcription of p21 and higher levels of Sox2 and c-Myc under the condition of 4E-BPs. Accordingly, the expression of exogenous Oct4 alone was sufficient to induce pluripotency in p53 and 4E-BP1/2 double deletion mutant fibroblasts.

## 4. Concluding Remarks

The establishment of iPSC methodology and dissection of the mechanisms regarding reprogramming are a milestone in the long journey of stem cell research both theoretically and practically, providing a sufficient tool with which to tackle fundamental biomedical questions regarding epigenetics-mediated (de)differentiation, as well as providing a valuable cell source for tissue regeneration, human disease modeling, and drug discovery. Due to the efforts made to improve the protocols for iPSC generation, particularly with regard to patient-specific somatic cells, with a focus on increased efficiency and safety, significant progress has been made by employing a variety of (epi)genetic and biochemical approaches. In addition, the molecular mechanisms behind reprogramming have been extensively studied at biochemical, genetic, and epigenetic levels. However, technical challenges in the generation of iPSCs and safety concerns for the use of iPSCs in clinical applications remain big issues which require solving.

Although various strategies have been invented to enhance reprogramming efficiency and to improve the issue of safety, regrettably, none of these strategies could ensure both the high generation efficiency and safety of iPSCs. For example, while virus-mediated ectopic expression of the core reprogramming factors or knockdown of key epigenetic factors led to high generation efficiency, the integration of virus vectors could lead to tumorigenesis. Likewise, some strategies without virus vector meditation such as virus vector-free or transgene-free methods, such as the modified mRNA strategy and small molecule treatments to inhibit some barriers or activate enhancers of the reprogramming process, can improve the safety of iPSCs but their efficiency is much lower than that conferred by the virus- or other transgene-mediated methods. In addition, some small molecules may have multiple targets and thus come with the possibility of off-target effects which could lead to the unpredictable quality and safety concerns for the generated iPSCs. Thus, it is of great importance to carry out high-throughput chemical screens, transcriptomic, and proteomic studies so that more small molecules, chromatin remodelers, and other epigenetic modifiers can be identified and employed to enhance iPSC generation efficiency without raising safety and quality issues.

On the other hand, mechanism studies may facilitate the discovery of new strategies focusing on different targets to significantly enhance reprogramming efficiency. Previous studies have made this approach applicable, such as those leading to the discovery of epigenetic barriers to the reprogramming. By removing these barriers, reprogramming efficiency could be dramatically promoted. Although several barriers have been identified, more efforts are required to unveil new barriers and activators.

Alternatively, microRNA approaches could be also applied for the dissection of pathways implicated in iPSC reprogramming to further understand the crosstalk among metabolic pathways and the molecular agents known to serve as (epi)genetic modifiers or drivers. Further understanding of reprogramming mechanisms and development of safer and more efficient reprogramming strategies will benefit biomedical studies as well as iPSC-mediated regeneration medicine and transplant therapy.
